# To Evaluate Whether Pretreatment CA19‐9 and DUPAN‐2 Levels Can Serve as Predictive Markers to Guide the Choice Between NAT and Upfront Surgery in Pancreatic Cancer

**DOI:** 10.1002/ags3.70140

**Published:** 2025-11-28

**Authors:** Hiromichi Kawaida, Wataru Izumo, Ryo Saito, Hidetake Amemiya, Suguru Maruyama, Katsutoshi Shoda, Kensuke Shiraishi, Shinji Furuya, Yoshihiko Kawaguchi, Daisuke Ichikawa

**Affiliations:** ^1^ First Department of Surgery; Faculty of Medicine University of Yamanashi Yamanashi Japan

**Keywords:** early recurrence, neoadjuvant chemotherapy, pancreatic cancer, tumor marker, upfront surgery

## Abstract

**Aim:**

Pancreatic cancer (PC) remains one of the most lethal malignancies, with early recurrence severely affecting prognosis even after curative resection. While neoadjuvant treatments (NAT), such as gemcitabine plus S‐1 (NAT‐GS), have improved outcomes, predicting their effectiveness and optimizing patient selection remain challenging. This study aimed to investigate whether pretreatment CA19‐9 and DUPAN‐2 levels can serve as predictive markers to guide the choice between NAT and upfront surgery in pancreatic cancer.

**Methods:**

We retrospectively analyzed 190 patients with PC (67 with NAT‐GS and 123 with upfront surgery). Patients were stratified into three groups based on their pre‐NAT CA19‐9/DUPAN‐2 levels: both within the reference range, one exceeding, or both exceeding. The 12 month recurrence‐free survival (12MRFS) rates were assessed using the Kaplan–Meier method, with log‐rank and other appropriate statistical tests for comparison.

**Results:**

NAT‐GS was significantly more effective only in patients with one tumor marker above the reference range, comparing those who achieved 12 month MRFS with those who did not. In the Kaplan–Meier curve, among patients with only one elevated tumor marker, the NAT‐GS group showed a significantly better 12MRFS ratio than the upfront surgery group (78.6% vs. 51.2%, *p* = 0.034).

**Conclusion:**

The combination of pre‐NAT CA19‐9 and DUPAN‐2 levels may serve as a useful criterion for selecting between upfront surgery, NAT‐GS, or more intensive chemotherapy. This suggests the possibility of a tailored approach, in which a combination of tumor markers would determine whether to opt for upfront surgery, NAT‐GS, or more intensive chemotherapy.

## Graphical abstract

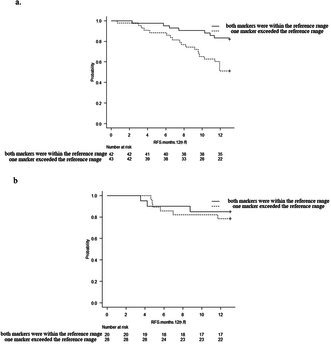

## 1 Introduction

Pancreatic cancer (PC) is one of the most lethal and aggressive malignant tumors [1]. Moreover, only 10%–20% of patients are diagnosed with resectable PC, among whom 50%–86% experience tumor recurrence despite curative resection [2, 3, 4]. Furthermore, up to 35% of patients experience disease recurrence within 12 months after surgery, and the poor prognosis due to early recurrence has raised concerns regarding the effectiveness of pancreatectomy as the main treatment strategy [5].

Various neoadjuvant treatments (NAT) have been used to improve the prognosis of patients with resectable PC. However, NAT conferred no survival benefit compared with upfront surgery [6, 7, 8, 9]. In Japan, a randomized phase III trial (Prep‐02/JSAP‐05) successfully demonstrated the clinical benefit of NAT on overall survival (OS) through an intention‐to‐treat analysis [10]. Based on these findings, Japanese clinical practice guidelines recommend gemcitabine plus S‐1 combination therapy (GS) as NAT (NAT‐GS). Although this treatment improves the median OS to 36.7 months [10], the prognosis remains poor. In cases in which NAT‐GS is insufficient, it is necessary to establish an alternative treatment strategy.

Predicting the therapeutic effect of NAT before its administration may help determine the indications for upfront surgery and assist in selecting an appropriate NAT regimen [11]. Radiographic staging can only detect visible disease, and the therapeutic effect of NAT may not be reflected in the radiographic response [11]. Although tumor markers have the advantage of being disease‐specific, prognostic prediction using tumor markers before NAT remains unclear [11]. In particular, NAT is expected to have limited efficacy in cases of early recurrence. However, there are patients with a low likelihood of recurrence in whom NAT may be unnecessary, making them suitable candidates for upfront surgery.

Several studies have reported that patients in whom all tumor markers remain within the reference range have a more favorable prognosis compared with those in whom at least one marker exceeds the reference range [12, 13].

Therefore, from the perspective of early recurrence‐free survival, we evaluated whether stratifying patients using multiple tumor markers measured prior to NAT could effectively distinguish cases in which NAT improves prognosis from cases in which its efficacy is limited.

## 2 Methods

### 2.1 Patient Enrollment

We retrospectively investigated the data of 190 patients who underwent curative resection of pancreatic adenocarcinoma at our institution between January 2013 and December 2023. All patients had a histologically confirmed pancreatic ductal adenocarcinoma. We included patients who were aged ≥ 18 years and had an Eastern Cooperative Oncology Group performance status of 0 or 1. Patients whose serum carcinoembryonic antigen (CEA), carbohydrate antigen (CA 19–9), and Duke pancreatic monoclonal antigen type 2 (DUPAN‐2) levels were not examined before neoadjuvant therapy were excluded.

Resectable PC was determined based on computed tomography (CT) and magnetic resonance imaging (MRI) examinations performed before NAT, in accordance with the National Comprehensive Cancer Network Guidelines Version 2 [14].

This study was approved by the Institutional Review Board of the Faculty of Medicine, University of Yamanashi (No. H30186).

### 2.2 Clinical Examination and Neoadjuvant Chemotherapy

We investigated age, sex, tumor location, and laboratory data. The laboratory data included serum CEA, CA19‐9, and DUPAN‐2 levels; inflammatory markers such as the lymphocyte‐to‐monocyte ratio (LMR), neutrophil‐to‐lymphocyte ratio, lymphocyte‐to‐C‐reactive protein ratio (LCR), and platelet‐to‐lymphocyte ratio; and prognostic nutritional index as a nutritional marker.

For patients who underwent NAT (as defined previously), data collected before NAT were used for analysis. Data collected immediately before surgery were used for patients who did not undergo NAT. Laboratory data were based on measurements obtained after jaundice reduction via endoscopic stent placement or percutaneous transhepatic drainage, particularly if the serum bilirubin levels were elevated. In such cases, the values at the point when the bilirubin level reached ≤ 2 mg/dL were selected.

NAT was introduced in September 2019. Patients diagnosed with adenocarcinoma based on histology or cytology before undergoing NAT were considered eligible. The treatment criteria for patients were Eastern Cooperative Oncology Group (ECOG) performance status (PS) < 2, adequate hematological, hepatic, renal, cardiac, and respiratory function.

The NAT regimen consisted of intravenous gemcitabine (1000 mg/m^2^ on days 1 and 8 of a 21 d cycle) plus oral S‐1 (80, 100, or 120 mg/d according to the body surface area, administered on days 1–14 of a 21 d cycle). This regimen was repeated for a total of 2 cycles [10].

### 2.3 Adjuvant Therapy

As adjuvant therapy, patients with resected PC received either S‐1 orally twice daily for 28 d (followed by a 14 d rest period, completing a 6 week cycle for up to 4 cycles) [15] or intravenous gemcitabine administered on days 1, 8, and 15 (completing a 4 week cycle for up to 6 cycles) [16]. Patients with renal dysfunction or interstitial pneumonia were excluded.

### 2.4 Follow‐Up Protocol

For all patients, postoperative follow‐up included monthly blood tests with tumor markers (CEA, CA19‐9, and DUPAN‐2) and CT scans from the chest to the pelvis performed every 3 months.

### 2.5 Evaluation Factor

In this study, according to research establishing “12 months” as the optimal cutoff value for early recurrence [17], we investigated the relationship between tumor markers measured before surgery or NAT and the 12 month recurrence‐free survival rate (12MRFSR). Tumor markers were classified into three groups based on reference values using CA19‐9 and DUPAN‐2 [12, 13]. The 12 month recurrence‐free survival (12MRFS) was calculated from the date of surgery until documented recurrence. Recurrence was diagnosed using various imaging modalities, specifically CT, MRI, or positron emission tomography.

Initially, chi‐square tests were applied as a preliminary comparison of 12 month recurrence‐free survival (12MRFS) between groups. Since RFS is a time‐to‐event variable, Kaplan–Meier curves with the log‐rank test were subsequently used as the primary method to evaluate group differences.

### 2.6 Statistical Analysis

Fisher's exact test, Mann–Whitney U test, and Student's t‐test were used for statistical analyses. Notably, 12MRFS was estimated using the Kaplan–Meier method, and survival curves were compared using the log‐rank test. All statistical analyses were conducted using EZR (Saitama Medical Center, Jichi Medical University, Saitama, Japan), which serves as the graphical user interface for R software (R Foundation for Statistical Computing, Vienna, Austria).

## 3 Results

### 3.1 Patient Characteristics

In total, 190 patients who underwent resection for resectable PC during the specified period were included in this study. Among them, 67 underwent NAT‐GS (NAT‐GS group) and 123 underwent upfront surgery (upfront surgery group).

There were no significant differences in the age or sex ratios between the groups. Albumin levels were higher in the upfront surgery group than in the NAT‐GS group, whereas total cholesterol levels were higher in the NAT‐GS group than in the upfront surgery group. Among the inflammatory markers, LMR and LCR were significantly higher in the NAT‐GS group than in the upfront surgery group. Tumor marker analysis revealed that CEA levels were within the reference range in both groups, whereas CA19‐9 and DUPAN‐2 levels exceeded the reference range in both groups.

Early recurrence within 12 months was observed in 47 and 17 patients in the upfront surgery and NAT‐GS groups, respectively. No significant differences were observed between the two groups (*p* = 0.06) (Table 1).

**TABLE 1:** Baseline characteristics.

	Upfront surgery group	NAT‐GS group	*p*
Number	123	67	
Age median (range)	71.0 (42–84)	70.7 (46–84)	0.79
Male	77	38	0.44
Female	46	29	
Location			
Ph	73	33	0.22
Pb, Pt	40	34	
Laboratory data			
Albumin g/dL	4.0 ± 0.5	3.9 ± 0.4	0.042
Total cholesterol g/dL	186.7 ± 37.8	198.5 ± 50.6	0.073
Lymphocyte /μL	1380 ± 510	1500 ± 590	0.14
Inflammatory biomarker			
NLR	2.9 ± 2.5	2.7 ± 1.7	0.5
LMR	4.5 ± 1.8	5.5 ± 2.8	0.003
LCR	9380 ± 6070	12 190 ± 13 100	0.045
PLR	182.4 ± 169.8	174.5 ± 91.8	0.73
Nutritional marker			
PNI	47.3 ± 5.6	46.5 ± 5.0	0.35
Tumor markers			
CEA ng/ml	4.5 ± 4.9	3.7 ± 3.0	0.26
CA19‐9 U/mL	235.3 ± 494.3	171.9 ± 300.0	0.34
DuPAN‐2 U/ml	342.0 ± 513.5	351.1 ± 512.9	0.91
Intraoperative findings			
TP	2	2	0.5
PD	72	32	
DP	49	33	
Blood loss (ml)	834 ± 559	561 ± 562	0.002
Duration of operation (min)	474 ± 131	494 ± 127	0.3
Pathological findings			
Tumor size (mm)	25 ± 11	21 ± 8	0.018
Lymph node metastasis			
Yes	68	33	0.45
No	55	34	
Pancreatic cut margin			
Positive	4	6	0.17
Negatine	119	61	
Adjuvant chemotherapy			
Yes	94	57	0.19
No	29	10	
12MRFS			
Achieved	74	50	0.06
Not achieved	49	17	

Abbreviations: 12MRFS, 12‐month recurrence‐free survival; CA19‐9, carbohydrate antigen 19–9; CEA, carcinoembryonic antigen; DP, Distal pancreatectomy; DUPAN‐2, Duke pancreatic monoclonal antigen type 2; LCR, Lymphocyte‐to‐C‐Reactive Protein ratio; LMR, Lymphocyte‐to‐monocyte ratio; NAT‐GS, neoadjuvant treatments gemcitabine plus S‐1; NLR, Neutrophil‐to‐lymphocyte ratio; Pb, Pancreatic body; PD, Pancreatoduodenectomy; Ph, Pancreatic head; PLR, Platelet‐to‐lymphocyte ratio; PNI, Prognostic nutritional index; Pt, Pancreatic tail; TP, Total pancreatectomy.

### 3.2 Intraoperative Findings and Adjuvant Chemotherapy

Surgical procedures were not significantly different between the two groups. However, blood loss was significantly greater in the upfront surgery group than in the NAT‐GS group. Tumor size was significantly smaller in the NAC‐GS group, but no significant difference was observed in the lymph node metastasis rate. No significant differences were observed between the groups in the proportions of patients who received adjuvant therapy (Table 1).

### 3.3 Comparison of Early Recurrence Rates Based on Tumor Markers

We excluded CEA from our primary analysis because its values were generally within the normal range in both groups, thus limiting its utility for stratification. Cases in which CA19‐9 and DUPAN‐2 levels exceeded the reference range were categorized into three groups: cases where both tumor markers were within the reference range, cases where one tumor marker exceeded the reference range, and cases where both tumor markers exceeded the reference range.

In cases where one tumor marker exceeded the reference range, univariate analysis showed that only NAT‐GS and the absence of lymph node metastasis were significantly correlated with achieving 12 month RFS (Table 2). In multivariate analysis, these two factors remained significant, with NAT‐GS being the only preoperative factor that independently predicted the achievement of 12 month RFS (Table 3).

**TABLE 2:** Characteristics in cases in which only one tumor marker exceeded the reference range.

12MRFS	Achieved	Not achieved	*p*
Number	44	27	
Age median (range)	70.7 (48–83)	73.0 (46–84)	0.24
Male	31	17	0.6
Female	13	10	
Location			
Ph	23	16	0.63
Pb, Pt	21	11	
Pre‐NAT laboratory data			
Albumin g/dL	4.0 ± 0.4	4.0 ± 0.4	0.817
Total cholesterol g/dL	196.2 ± 49.5	176.1 ± 38.1	0.07
Lymphocyte /μL	1490 ± 530	1310 ± 530	0.17
Inflammatory biomarker			
NLR	2.7 ± 2.2	2.7 ± 1.5	0.99
LMR	5.3 ± 2.0	5.3 ± 3.6	0.99
LCR	13 400 ± 15 310	9280 ± 6670	0.19
PLR	160.6 ± 91.0	176.8 ± 81.0	0.45
Nutritional marker			
PNI	47.6 ± 5.1	46.9 ± 4.8	0.58
Tumor markers			
CEA ng/ml	4.2 ± 2.9	3.4 ± 1.8	0.26
CA19‐9 U/ml	132.5 ± 228.8	116.9 ± 184.5	0.77
DuPAN‐2 U/ml	275.6 ± 454.9	226.5 ± 454.8	0.66
Preoperative data			
Total cholesterol g/dL	187.6 ± 39.0	171.9 ± 28.5	0.08
CEA ng/ml	4.4 ± 2.9	3.5 ± 2.0	0.17
CA19‐9 U/ml	80.8 ± 144.7	107.2 ± 189.2	0.52
DuPAN‐2 U/ml	232.5 ± 416.8	205.7 ± 431.1	0.8
Upfront surgery	22	21	0.025
NAT‐GS	22	6	
Intraoperative findings			
TP	1	1	0.74
PD	21	15	
DP	22	11	
Blood loss (ml)	666 ± 493	677 ± 525	0.93
Duration of operation (min)	449 ± 98	489 ± 146	0.17
Pathological findings			
Tumor size (mm)	25 ± 16	31 ± 17	0.13
Lymph node metastasis			
Presence	16	22	< 0.001
Absence	28	5	
Pancreatic cut margin			
Positive	4	3	0.56
Negative	40	24	
Adjuvant chemotherapy			
Yes	39	21	0.31
No	5	6	

**TABLE 3:** Multivariate analysis in the group in which only one tumor marker exceeded the reference range.

	Odds ratio	95% CI	*p*
Pre‐NAT Total cholesterol	0.997	0.981–1.01	0.794
Upfront surgery or NAT‐GS	0.251	0.071–0.893	0.033
Lymph node metastasis	0.129	0.038–0.437	< 0.001
Preoperative total cholesterol	0.991	0.970–1.010	0.415

Abbreviation: CI, confidence interval.

In cases where both tumor markers were within the reference range, univariate analysis identified the absence of lymph node metastasis as the only significant factor, while no independent predictors were detected in multivariate analysis (File S2a). In cases where both tumor markers exceeded the reference range, CA19‐9 and DUPAN‐2 were significant in univariate analysis (File S2b); however, no independent predictors were identified in multivariate analysis.

### 3.4 12‐Month Recurrence‐Free Survival Rate

To more accurately assess the time‐to‐event variable, Kaplan–Meier analysis with the log‐rank test was performed.

**FIGURE 1:**
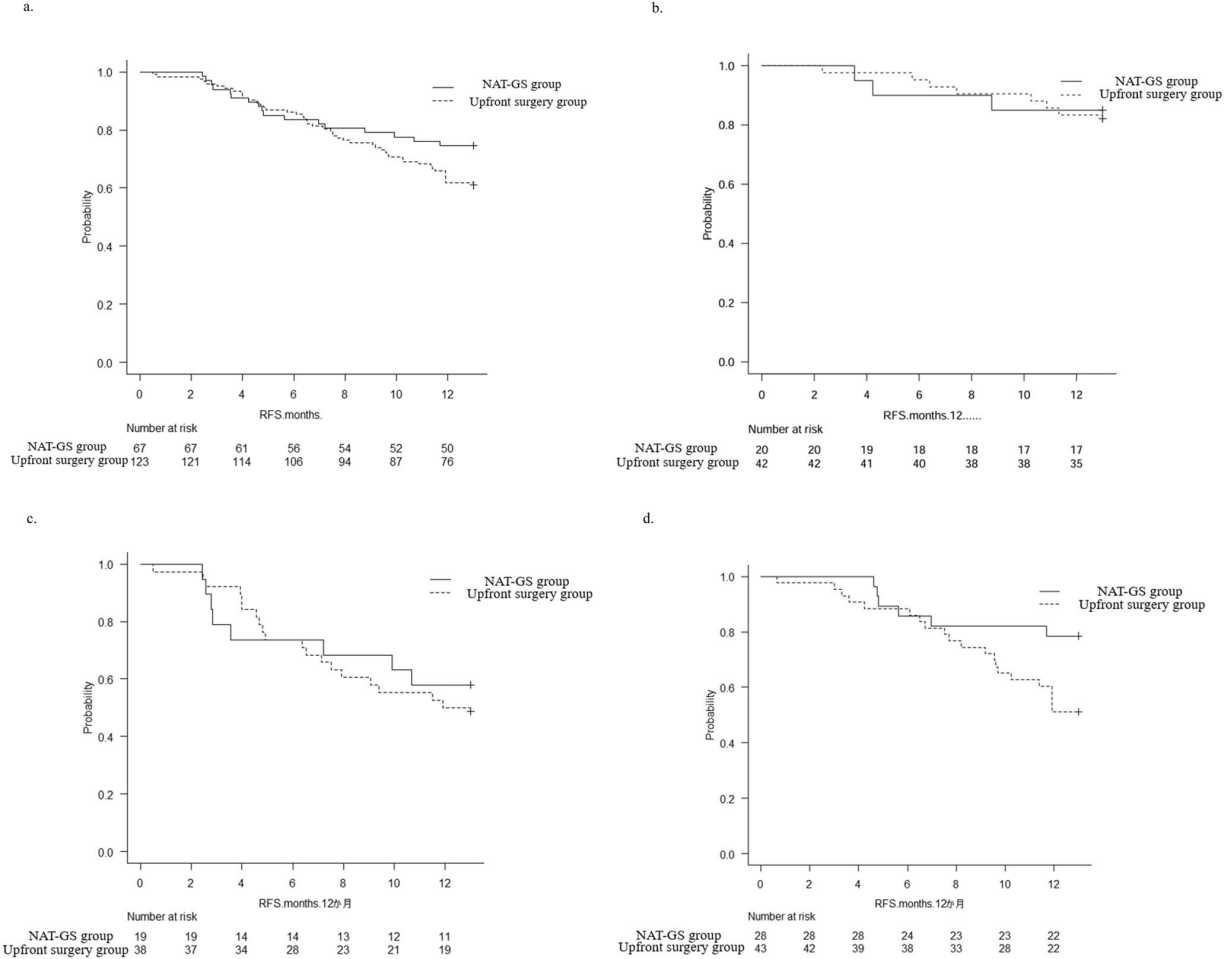
Comparison of recurrence‐free survival between the upfront and NAT‐GS groups. (a) Kaplan–Meier curve for the 12 month recurrence‐free survival rate (12MRFSR) in all cases in both the upfront surgery and NAT‐GS groups. This difference was not statistically significant between the two groups. (b) There was no significant difference between the two groups for cases in which both tumor markers were within the normal range. (c) No significant difference was observed in cases where both markers exceeded the reference range. (d) In cases where only one tumor marker exceeded the reference range, the NAT‐GS group showed a significantly better 12MRFSR than the upfront surgery group.

The Kaplan–Meier curve for the 12MRFSR in all cases for both the upfront surgery and NAT‐GS groups is shown in (Figure 1a). No significant differences were observed between the two groups.

In the cases in which both tumor markers were within the reference range, no significant differences were observed (85.0% vs. 83.3%, *p* = 0.75 Figure 1b). Similarly, no significant difference was observed in cases where both markers exceeded the reference range (57.9% vs. 50.0%, *p* = 0.59) (Figure 1c).

In contrast, in cases in which only one tumor marker exceeded the reference range, the NAT‐GS group showed significantly better 12MRFSR than the upfront surgery group (upfront surgery group vs. NAT‐GS group: 51.2% vs. 78.6%, *p* = 0.034) (Figure 1d).

**FIGURE 2:**
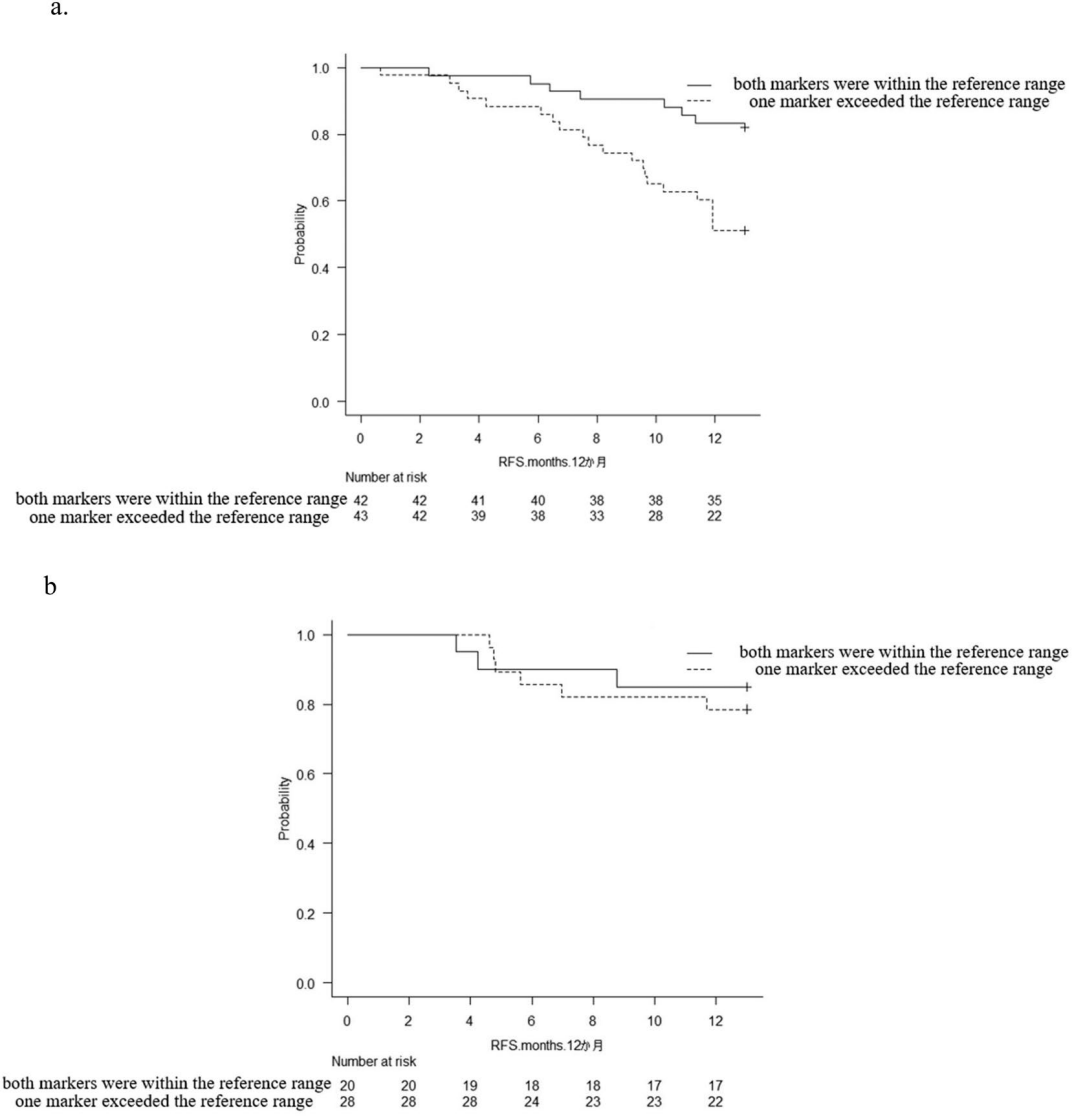
Comparison of recurrence‐free survival between cases where both tumor markers were within the normal range and cases where one tumor marker exceeded the reference range. (a) In the group that did not undergo NAT, the 12MRFSR was significantly worse in cases where one tumor marker exceeded the reference range than in cases where both tumor markers were within the reference range (*p* = 0.004). (b) After undergoing NAT, the 12MRFSR of the patients improved to a comparable level in both groups (*p* = 0.62).

In the group that did not undergo NAT, the 12MRFSR was significantly worse in cases where one tumor marker exceeded the reference range than in cases where both tumor markers were within the reference range (*p* = 0.004) (Figure 2a). However, after undergoing NAT, the 12MRFSR improved to a comparable level in both groups (*p* = 0.62) (Figure 2b).

## 4 Discussion

In the current study, we demonstrated the potential of combining two tumor markers to predict the impact of NAT on early recurrence after surgery. Our results showed that, in the comparison between the NAT and upfront surgery groups, the 12MRFSR was significantly improved only in cases where one tumor marker exceeded the reference range. This finding was also demonstrated using the Kaplan–Meier curve.

In the upfront surgery group, cases in which only one tumor marker exceeded the reference range had a significantly lower 12MRFSR than those in which both tumor markers were within the reference range. However, after undergoing NAT, there were no significant differences in the 12MRFSR between the two groups.

To prevent early recurrence, NAT may not be beneficial if we encounter cases in which both tumor markers are within the reference range. Conversely, in cases in which both tumor markers exceed the reference range, two courses of NAT‐GS may be insufficient. In such situations, it may be necessary to extend the duration of NAT or introduce a more intensive NAT regimen [18].

The relationship between PC prognosis and tumor markers has often been analyzed. Several studies have reported that post‐NAT CA19‐9 levels > 100 U/mL are associated with poor prognosis [9, 18, 19]. Several studies have also suggested that normalization of CA19‐9 levels after neoadjuvant therapy may serve as a favorable prognostic factor [11]. Moreover, reports suggest that decreased or normalized DUPAN‐2 levels after neoadjuvant therapy are associated with a reduced risk of postoperative recurrence [20, 21].

However, using only one tumor marker among multiple available markers may not be sufficient for prognostic prediction or treatment decisions. Several studies have reported that combining multiple tumor markers is associated with a more accurate prognostic evaluation [13]. Among the various combinations of tumor markers, the use of both CA19‐9 and DUPAN‐2 has been frequently investigated. Previous studies have reported that this combination is significantly associated with disease‐free survival and disease‐specific survival [22], and serves as a potential predictor of hematogenous metastasis occurring within 1 year [23]. Furthermore, Sumiyoshi et al. proposed that patients with PC who exhibit both markers within the normal reference range should be considered as having early‐stage disease [12]. However, most previous studies have used data obtained immediately before surgery. To the best of our knowledge, the present study is the first to evaluate the predictive factors for early recurrence based on pre‐NAT data.

Regarding CA19‐9, the influence of Lewis antigen status is a known issue. There were six patients with CA19‐9 < 2 U/mL in the upfront surgery group and two patients in the NAT‐GS group. Approximately 4.1%–10.5% of the total population has the Lewis phenotype of erythrocytes, which cannot produce CA19‐9 [24, 25, 26]. Regarding the relationship between CA19‐9 and the Lewis antigen in patients with PC, Luo et al. reported that 8.4% of patients were Lewis antigen‐negative (Lewis (−)). However, only 41.9% of these Lewis (−) individuals had CA19‐9 levels ≤ 2 U/mL. Notably, CA19‐9 levels were elevated (> 37 U/mL) in 27.4% of Lewis (−) patients [26]. Based on these previous reports, the present study included patients with low CA19‐9 levels. For a more accurate evaluation, the Lewis antigen status should be assessed in all patients; however, this may be impractical.

Liquid biopsies, such as circulating tumor DNA and circulating tumor cells, which enable the real‐time monitoring of tumor dynamics, have gained increasing attention [27]. These biomarkers are also expected to serve as more accurate predictors of treatment efficacy in chemotherapy for patients with PC [28, 29]. However, owing to their low detection sensitivity and high cost, tumor markers continue to serve as the main criteria for clinical decision‐making in current practice.

This study had several limitations. First, this was a single‐center retrospective study. Second, our study is subject to temporal bias, since the upfront surgery cohort includes patients from 2013, whereas the NAT cohort only begins in 2019. Additionally, it should be noted that patients who experienced disease progression during NAT and subsequently dropped out before surgery may have been excluded from the NAT‐GS group. Third, the limited number of patients in the NAT group reduced the power of the subgroup analyses, making it difficult to draw definitive conclusions. Fourth, although some patients in this cohort may have been Lewis antigen‐negative, we were unable to assess Lewis antigen status in this study. Such patients were presumed to be included in groups where both tumor markers were within the reference range and one tumor marker exceeded the reference range. To clarify the significance of neoadjuvant therapy in patients with resectable PC, future prospective studies with well‐balanced patient enrollment across subgroups comparing NAT and upfront surgery are warranted.

## 5 Conclusions

The combination of pre‐NAT CA19‐9 and DUPAN‐2 levels may serve as a useful criterion for selecting between upfront surgery, NAT‐GS, or more intensive chemotherapy as therapeutic strategies for PC. However, as this study was limited by its single‐center retrospective design, the potential effects observed require confirmation in well‐designed prospective studies.

## Author Contributions

**Hiromichi Kawaida:** conceptualization, methodology, data curation, investigation, validation, formal analysis, writing – original draft, writing – review and editing, supervision. **Wataru Izumo:** investigation, formal analysis, supervision. **Ryo Saito:** investigation. **Hidetake Amemiya:** investigation. **Suguru Maruyama:** investigation. **Katsutoshi Shoda:** investigation. **Kensuke Shiraishi:** investigation. **Shinji Furuya:** investigation. **Yoshihiko Kawaguchi:** investigation. **Daisuke Ichikawa:** supervision.

## Funding

The authors have nothing to report.

## Ethics Statement

This study was reviewed and approved by the ethics committee of the University of Yamanashi.

## Conflicts of Interest

Daisuke Ichikawa is an Editorial Board Member of Annals of Gastroenterological Surgery. He was not involved in the editorial process or the peer review of this manuscript. The other authors have no conflicts of interest to declare.

## Supporting information


**Data S1:** Supplementary Tables.

## Data Availability

The authors have nothing to report.

## References

[1] R. L. Siegel , K. D. Miller , and A. Jemal , “Cancer Statistics, 2018,” CA: A Cancer Journal for Clinicians 68 (2018): 7–30. 29313949 10.3322/caac.21442

[2] M. H. Katz , G. R. Varadhachary , J. B. Fleming , et al., “Serum CA 19‐9 as a Marker of Resectability and Survival in Patients With Potentially Resectable Pancreatic Cancer Treated With Neoadjuvant Chemoradiation,” Annals of Surgical Oncology 17, no. 7 (2010): 1794–1801. 20162463 10.1245/s10434-010-0943-1 PMC2889288

[3] U. K. Ballehaninna and R. S. Chamberlain , “The Clinical Utility of Serum CA 19‐9 in the Diagnosis, Prognosis and Management of Pancreatic Adenocarcinoma: An Evidence Based Appraisal,” J Gastrointest Oncol 3 (2012): 105–119. 22811878 10.3978/j.issn.2078-6891.2011.021 PMC3397644

[4] T. M. Bauer , B. F. El‐Rayes , X. Li , et al., “Carbohydrate Antigen 19‐9 Is a Prognostic and Predictive Biomarker in Patients With Advanced Pancreatic Cancer Who Receive Gemcitabine‐Containing Chemotherapy: A Pooled Analysis of 6 Prospective Trials,” Cancer 119 (2013): 285–292. 22786786 10.1002/cncr.27734 PMC4261189

[5] R. P. Jones , E. E. Psarelli , R. Jackson , et al., “Patterns of Recurrence After Resection of Pancreatic Ductal Adenocarcinoma: A Secondary Analysis of the ESPAC‐4 Randomized Adjuvant Chemotherapy Trial,” JAMA Surgery 154, no. 11 (2019): 1038–1048. 31483448 10.1001/jamasurg.2019.3337 PMC6727687

[6] J. Y. Jang , Y. Han , H. Lee , et al., “Oncological Benefits of Neoadjuvant Chemoradiation With Gemcitabine Versus Upfront Surgery in Patients With Borderline Resectable Pancreatic Cancer: A Prospective, Randomized, Open‐Label, Multicenter Phase 2/3 Trial,” Annals of Surgery 268, no. 2 (2018): 215–222. 29462005 10.1097/SLA.0000000000002705

[7] P. Ghaneh , D. Palmer , S. Cicconi , et al., “Immediate Surgery Compared With Short‐Course Neoadjuvant Gemcitabine Plus Capecitabine, FOLFIRINOX, or Chemoradiotherapy in Patients With Borderline Resectable Pancreatic Cancer (ESPAC5): A Four‐Arm, Multicentre, Randomised, Phase 2 Trial,” Lancet Gastroenterology & Hepatology 8, no. 2 (2023): 157–168. 36521500 10.1016/S2468-1253(22)00348-X

[8] Y. Yabushita , R. Matsuyama , K. Miyake , et al., “Outcomes of Neoadjuvant Gemcitabine Plus S‐1 and Radiation Therapy for Borderline Resectable Pancreatic Cancer,” Journal of Hepato‐Biliary‐Pancreatic Sciences 30, no. 4 (2023): 493–502. 36178433 10.1002/jhbp.1245

[9] S. Yamada , D. Hashimoto , T. Yamamoto , et al., “Reconsideration of the Clinical Impact of Neoadjuvant Therapy in Resectable and Borderline Resectable Pancreatic Cancer: A Dual‐Institution Collaborative Clinical Study,” Pancreatology 24, no. 4 (2024): 592–599. 38548551 10.1016/j.pan.2024.03.012

[10] F. Motoi , A. Kudo , K. Okada , et al., “Neoadjuvant Chemotherapy With Gemcitabine and S‐1 Versus Upfront Surgery for Resectable Pancreatic Cancer: Results of the Randomized Phase II/III Prep‐02/JSAP05 Trial,” Annals of Surgery 280, no. 5 (2024): 887–896. 40235447 10.1097/SLA.0000000000006730 PMC12695294

[11] T. Sumiyoshi , K. Uemura , R. Shintakuya , et al., “Clinical Utility of the Combined Use of CA19‐9 and DUPAN‐2 in Pancreatic Adenocarcinoma,” Annals of Surgical Oncology 31, no. 7 (2024): 4665–4672. 38652196 10.1245/s10434-024-15221-z PMC11164736

[12] M. Aldakkak , K. K. Christians , A. N. Krepline , et al., “Pre‐Treatment Carbohydrate Antigen 19–9 Does Not Predict the Response to Neoadjuvant Therapy in Patients With Localized Pancreatic Cancer,” HPB: The Official Journal of the Hepato‐Pancreato‐Biliary Association 17 (2015): 942–952. 10.1111/hpb.12448 PMC4571763 26255895

[13] M. Nagai , K. Nakamura , T. Terai , et al., “Significance of Multiple Tumor Markers Measurements in Conversion Surgery for Unresectable Locally Advanced Pancreatic Cancer,” Pancreatology 23, no. 6 (2023): 721–728. 37328387 10.1016/j.pan.2023.06.001

[14] “NCCN Clinical Practice Guidelines in Oncology (NCCN Guidelines). NCCN,” (2022).

[15] K. Uesaka , N. Boku , A. Fukutomi , et al., “Adjuvant Chemotherapy of s‐1 Versus Gemcitabine for Resected Pancreatic Cancer: A Phase 3, Open‐Label, Randomised, Non‐Inferiority Trial (Jaspac 01),” Lancet 388, no. 10041 (2016): 248–257. 27265347 10.1016/S0140-6736(16)30583-9

[16] H. A. Burris, 3rd , M. J. Moore , J. Andersen , et al., “Improvements in Survival and Clinical Benefit With Gemcitabine as First‐Line Therapy for Patients With Advanced Pancreas Cancer: A Randomized Trial,” Journal of Clinical Oncology 15, no. 6 (1997): 2403–2413. 9196156 10.1200/JCO.1997.15.6.2403

[17] A. I. Ekkebus , I. Q. Molenaar , E. Bastiaannet , et al., “Defining and Predicting Early Recurrence in 957 Patients With Resected Pancreatic Ductal Adenocarcinoma,” Annals of Surgery 269, no. 6 (2019): 1154–1162. 31082915 10.1097/SLA.0000000000002734 PMC6191366

[18] E. Waugh , J. Glinka , D. Breadner , et al., “Survival Benefit of Neoadjuvant FOLFIRINOX for Patients With Borderline Resectable Pancreatic Cancer,” Ann Hepatobiliary Pancreat Surg 28, no. 2 (2024): 229–237. 38296221 10.14701/ahbps.23-107 PMC11128787

[19] N. Kondo , K. Uemura , T. Sumiyoshi , et al., “Identification of Preoperative Risk Factors for Poor Survival in Patients With Resectable Pancreatic Cancer Treated With Upfront Surgery,” Digestive Surgery 38, no. 5–6 (2021): 352–360. 34689146 10.1159/000520064

[20] A. Sasaki , S. Inokuchi , S. Tsutsumi , et al., “Elevated Preoperative DUPAN‐2 Level Predicts Locoregional Recurrence After Pancreatectomy in Patients With Pancreatic Ductal Adenocarcinoma,” Anticancer Research 42, no. 4 (2022): 2071–2078. 35347030 10.21873/anticanres.15688

[21] Y. Sunagawa , S. Yamada , Y. Sato , et al., “Novel Prognostic Implications of DUPAN‐2 in the Era of Initial Systemic Therapy for Pancreatic Cancer,” Annals of Surgical Oncology 27, no. 6 (2020): 2081–2089. 31673938 10.1245/s10434-019-07981-w

[22] Y. Shimizu , T. Sugiura , R. Ashida , et al., “ASO Author Reflections: Prognostic Role of Preoperative Duke Pancreatic Monoclonal Antigen Type 2 Values in Patients With Pancreatic Cancer: Focusing on the Usefulness in Patients With Normal CA19‐9,” Annals of Surgical Oncology 30, no. 9 (2023): 5801–5802. 37355518 10.1245/s10434-023-13629-7

[23] H. Kurahara , K. Maemura , Y. Mataki , et al., “Clinical Significance of Serum Carbohydrate Antigen 19.9 and Duke Pancreatic Monoclonal Antigen Type 2 for the Prediction of Hematogenous Metastases in Patients With Pancreatic Ducal Adenocarcinoma,” Pancreatology 16, no. 6 (2016): 1051–1056. 27693096 10.1016/j.pan.2016.09.014

[24] A. Oba , C. Croce , P. Hosokawa , et al., “Prognosis Based Definition of Resectability in Pancreatic Cancer: A Road Map to New Guidelines,” Annals of Surgery 275, no. 1 (2022): 175–181. 32149822 10.1097/SLA.0000000000003859

[25] A. V. Hayman , S. J. Stocker , M. S. Baker , et al., “CA 19‐9 Nonproduction Is Associated With Poor Survival After Resection of Pancreatic Adenocarcinoma,” American Journal of Clinical Oncology 37, no. 6 (2014): 550–554. 23428954 10.1097/COC.0b013e318280d5f0

[26] G. Luo , Z. Fan , H. Cheng , et al., “New Observations on the Utility of CA19‐9 as a Biomarker in Lewis Negative Patients With Pancreatic Cancer,” Pancreatology 18, no. 8 (2018): 971–976. 30131287 10.1016/j.pan.2018.08.003

[27] K. C. S. Oliveira , I. B. Ramos , J. M. C. Silva , et al., “Current Perspectives on Circulating Tumor DNA, Precision Medicine, and Personalized Clinical Management of Cancer,” Molecular Cancer Research 18, no. 4 (2020): 517–528. 31996469 10.1158/1541-7786.MCR-19-0768

[28] M. Nagai , M. Sho , T. Akahori , K. Nakagawa , and K. Nakamura , “Application of Liquid Biopsy for Surgical Management of Pancreatic Cancer,” Ann Gastroenterol Surg 4, no. 3 (2020): 216–223. 32490335 10.1002/ags3.12317 PMC7240145

[29] S. Nishiwada , M. Sho , Y. Cui , et al., “A Gene Expression Signature for Predicting Response to Neoadjuvant Chemoradiotherapy in Pancreatic Ductal Adenocarcinoma,” International Journal of Cancer 148, no. 3 (2021): 769–779. 32895958 10.1002/ijc.33284 PMC8221275

