# Supplementary material for: To Evaluate Whether Pretreatment CA19‐9 and DUPAN‐2 Levels Can Serve as Predictive Markers to Guide the Choice Between NAT and Upfront Surgery in Pancreatic Cancer
Source: Ann Gastroenterol Surg. 2025 Nov 28;10(3):835–42. doi: 10.1002/ags3.70140 (PMC13178265; doi:10.1002/ags3.70140)
Supplement: Supplementary file 1 — Data S1: Supplementary Tables. [file AGS3-10-835-s001.docx]

Supplement Data

Table 2a. Characteristics in cases in which both tumor markers were within the reference range

| 12MRFS | achieved | not achieved | p Value |
| --- | --- | --- | --- |
| Number | 51 | 11 |  |
| Age median (range) | 69 (85-81) | 73 (46-83) | 0.25 |
| Male | 26 | 4 | 0.51 |
| Female | 25 | 7 |  |
| Location |  |  |  |
| Ph | 26 | 3 | 0.19 |
| Pb,Pt | 25 | 8 |  |
| Pre NAT Laboratory data |  |  |  |
| Albumin g/dL | 4.1±0.2 | 3.9±0.5 | 0.13 |
| Total cholesterol g/dL | 190±33 | 192±39 | 0.81 |
| Lymphocyte /μL | 1380±530 | 1480±640 | 0.59 |
| Inflammatory biomarker |  |  |  |
| NLR | 3.3±3.0 | 3.6±2.9 | 0.75 |
| LMR | 4.3±1.8 | 4.3±1.8 | 0.95 |
| LCR | 9740±5720 | 9590±8830 | 0.94 |
| PLR | 210±211 | 191±111 | 0.81 |
| Nutritional marker |  |  |  |
| PNI | 48±4 | 46±7 | 0.35 |
| Tumor markers |  |  |  |
| CEA ng/ml | 2.6±2.2 | 2.3±1.8 | 0.59 |
| CA19-9 U/ml | 16.1±9.6 | 17.1±8.9 | 0.77 |
| DuPAN-2 U/ml | 30.4±11.0 | 35.0±28.1 | 0.39 |
| Preoperative data |  |  |  |
| Total cholesterol g/dL | 187±34 | 183±49 | 0.75 |
| CEA ng/ml | 2.8±2.5 | 2.2±1.8 | 0.49 |
| CA19-9 U/ml | 15.8±9.1 | 15.3±9.2 | 0.85 |
| DuPAN-2 U/ml | 31±13 | 33±26 | 0.69 |
| Upfront surgery | 34 | 8 | 0.97 |
| NAT-GS | 17 | 3 |  |
| Intra operative findings |  |  |  |
| TP | 1 | 0 | 0.59 |
| PD | 28 | 3 |  |
| DP | 22 | 8 |  |
| Blood loss (ml) | 528±381 | 741±736 | 0.17 |
| Duration of operation (min) | 463±133 | 455±124 | 0.86 |
| Pathological findings |  |  |  |
| Tumor size (mm) | 21±10 | 24±7 | 0.25 |
| Lymph node metastasis |  |  |  |
| presence | 12 | 7 | 0.03 |
| absence | 39 | 4 |  |
| Pancreatic cut margin |  |  |  |
| positive | 1 | 0 | 1 |
| negative | 50 | 11 |  |
| Adjuvant chemotherapy |  |  |  |
| Yes | 42 | 8 | 0.43 |
| No | 9 | 3 |  |

Table 2b. Characteristics in cases where both tumor markers exceeded the reference range.

| 12MRFS | achieved | not achieved | p Value |
| --- | --- | --- | --- |
| Number | 28 | 29 |  |
| Age median (range) | 71 (62-79) | 72±(55-84) | 0.75 |
| Male | 16 | 21 | 0.27 |
| Female | 12 | 8 |  |
| Location |  |  |  |
| Ph | 21 | 17 | 0.26 |
| Pb,Pt | 7 | 12 |  |
| Pre NAT Laboratory data |  |  |  |
| Albumin g/dL | 3.9±0.5 | 3.9±0.5 | 0.92 |
| Total cholesterol g/dL | 188±39 | 500±57 | 0.37 |
| Lymphocyte /μL | 1430±550 | 1470±540 | 0.82 |
| Inflammatory biomarker |  |  |  |
| NLR | 2.6±1.6 | 2.4±0.8 | 0.5 |
| LMR | 4.4±1.6 | 5.1±2.1 | 0.15 |
| LCR | 9830±630 | 8710±6170 | 0.49 |
| PLR | 180±110 | 150±40 | 0.21 |
| Nutritional marker |  |  |  |
| PNI | 46±7 | 46±6 | 0.99 |
| Tumor markers |  |  |  |
| CEA ng/ml | 6.7±7.3 | 6.1±5.3 | 0.73 |
| CA19-9 U/ml | 341.2±257.2 | 734.3±858.7 | 0.022 |
| DuPAN-2 U/ml | 641±498 | 936±576 | 0.043 |
| Preoperative data |  |  |  |
| Total cholesterol g/dL | 186±42 | 186±44 | 0.97 |
| CEA ng/ml | 6.0±6.8 | 6.3±5.4 | 0.87 |
| CA19-9 U/ml | 247.2±246.7 | 622.3±720.5 | 0.011 |
| DuPAN-2 U/ml | 523±479 | 906±606 | 0.012 |
| Upfront surgery | 20 | 18 | 0.58 |
| NAT-GS | 8 | 11 |  |
| Intra operative findings |  |  |  |
| TP | 0 | 1 | 0.49 |
| PD | 20 | 17 |  |
| DP | 8 | 11 |  |
| Blood loss (ml) | 0009±771 | 1007±558 | 0.99 |
| Duration of operation (min) | 509±149 | 531±120 | 0.56 |
| Pathological findings |  |  |  |
| Tumor size (mm) | 25±10 | 29±12 | 0.19 |
| Lymph node metastasis |  |  |  |
| presence | 22 | 22 | 0.832 |
| absence | 6 | 7 |  |
| Pancreatic cut margin |  |  |  |
| positive | 1 | 1 | 0.93 |
| negative | 27 | 28 |  |
| Adjuvant chemotherapy |  |  |  |
| Yes | 19 | 22 | 0.59 |
| No | 9 | 7 |  |
